# Nuclear factor Y, a key player in neuronal gene regulation

**DOI:** 10.1177/00368504241264998

**Published:** 2024-07-23

**Authors:** Pedro Moreira, Roger Pocock

**Affiliations:** Development and Stem Cells Program, Monash Biomedicine Discovery Institute and Department of Anatomy and Developmental Biology, 2541 Monash University, Melbourne, Victoria, Australia

**Keywords:** *Caenorhabditis elegans*, gene regulation, neurodevelopment, pan-neuronal gene, neuron-specific gene

## Abstract

Establishing a functional nervous system is a complex process requiring tightly controlled gene expression programs to achieve the correct differentiation of distinct neuronal subtypes. The molecular programs required for neurons to acquire neuron-type-specific, and core pan-neuronal features mostly rely on sequence-specific transcription factors (TFs), which recognize and bind to cis-regulatory motifs present in the promoters of target genes. Recently, we investigated the role and mode of action of the NF-Y complex, a ubiquitously expressed transcriptional master regulator, in the *Caenorhabditis elegans* nervous system. We found that NFYA-1 is a pervasive regulator of neuron-specific and pan-neuronal gene batteries that are essential for neuronal development and function. Furthermore, we concluded that NFYA-1 acts cell autonomously by either directly binding to conserved motifs in target gene promoter regions or indirectly by regulating other transcriptional regulators to fine-tune gene expression. However, further studies are required to fully define the impact of the NF-Y complex on nervous system regulatory networks and how NF-Y coordinates with other TFs in this regard.

## Transcription factor control of nervous system development

During development, neurons acquire specific and shared characteristics essential for their morphology and function. Hence, it is important to understand what molecules are required for the correct expression of neuronal gene batteries responsible for neuron-type-specific and pan-neuronal functions.^1^ Great efforts have been made, using the *Caenorhabditis elegans* model, to dissect molecular mechanisms involved in neuron-type-specific gene expression (e.g. specialized neuronal features such as neurotransmitter pathways, ion channels, neuropeptides, and signaling receptors).^2^ It is now well accepted that neuron-type-specific features are controlled by the activity of transcription factors (TFs) that bind specific *cis-*regulatory motifs.^3–6^ In contrast, the regulatory programs that control pan-neuronal gene batteries (e.g. synaptic vesicles and neuropeptide release) rely on combinations of parallel-acting TFs that bind to *cis-*regulatory motifs.^3^ For example, a recent study by Leyva-Diaz and Hobert showed that six members of the CUT homeobox family redundantly control pan-neuronal gene expression.^7^ Moreover, these CUT homeobox TFs act in parallel with terminal fate selectors to perform this function in specific neuronal types.^7^

Whether individual TFs can impact the expression of both neuron-type-specific and pan-neuronal gene expression has been understudied. This could be due to the number of complex interactions that ubiquitous TFs exhibit. In a recent study, we aimed to dissect the molecular programs governed by the nuclear factor-YA (NFYA-1) TF in the nervous system.^1^ Our work showed how a TF, NFYA-1, can be a regulator of neuron-specific and pan-neuronal gene expression. We explored how NFYA-1 can directly and indirectly fine-tune gene expression in different neuronal contexts by cooperating with other transcriptional regulatory molecules.

## NFY function during nervous system development

The NF-Y complex is a heterotrimeric protein composed of three different subunits, NF-YA, NF-YB, and NF-YC, which interact with each other through evolutionary conserved motifs called HAP2, HAP3, and HAP5 to regulate gene expression.^8^ The NF-YA subunit contains a DNA binding domain that recognizes specific regions in target gene promoters—a consensus CCAAT box usually located in the minor groove of DNA. CCAAT boxes are frequently found at −60 to −100 bp from the transcriptional start site, and around 30% of all eukaryotic promoters are directly regulated by the NF-Y complex.^9,10^ The NF-Y complex is crucial for early mouse development as NF-YA knockout causes embryonic lethality.^11^ Other studies demonstrated that NF-Y controls cell proliferation of embryonic stem cells by directly regulating housekeeping genes and controlling cell identity programs by binding to distal enhancers in collaboration with cell type-specific master TFs.^12,13^ More recent studies revealed that conditional depletion of NF-YA in murine postmitotic neurons causes degeneration, possibly due to dysregulation of endoplasmic reticulum homeostasis.^14^ Further, studies in *Drosophila* revealed that NF-YC represses a key zinc-finger TF Senseless in R7 photoreceptor neurons for correct neuronal targeting and synapse formation.^15^

Despite the previous studies described above, the full impact of NF-Y in controlling neuronal gene batteries is not well understood. In a forward genetic screen for loss of neuronal fate in *C. elegans*, we isolated a mutation in an NF-Y TF subunit. We found that NF-Y loss causes defective expression of neuron-type-specific and pan-neuronal features expressed in a pair of glutamatergic interneurons (PVQ neurons). Moreover, we showed that the NF-Y subunits acts cell autonomously to control the expression of PVQ-specific and pan-neuronal features. These interesting findings led us to question: (1) How does NFYA-1 regulate neuron-specific and pan-neuronal features? (2) Does NFYA-1 cooperate with other TFs to regulate pan-neuronal gene expression? and (3) Are these mechanisms conserved across species?

To dissect NFYA-1 regulation of neuronal expression, we analyzed whole-animal ChIP-Seq profiles of GFP-tagged NFYA-1 from the Encode consortium.^16^ This bioinformatic analysis revealed that 90% of NFYA-1 binding peaks are located between 0 and 3 kb upstream of the transcription start site, as previously described in mammals.^17^ Since we were interested in the regulation of both neuron-type-specific and pan-neuronal features, we randomly picked neuron-type-specific and pan-neuronal genes for analysis. Interestingly, this analysis showed a regulatory bias toward pan-neuronal regulation, with 15/15 genes harboring a ChIP peak for NFYA-1 in comparison with 1/15 neuron-type-specific genes (several of which are broadly expressed). These data suggested that NFYA-1 predominantly binds to the regulatory regions of pan-neuronal genes when compared to neuron-type-specific genes. Despite the ChIP-Seq data, our extensive gene reporter analysis observed NFYA-1-dependent regulation of some neuron-type-specific genes, such as *del-1*/DEG/ENaC, *eat-4/VGLUT, unc-25/GAD* and *unc-47/VGAT*. In contrast, other neuron-type-specific genes were unaffected (*acr-2/AChR, cho-1/ChT* and *unc-17/ChAT).* This analysis suggests that NFYA-1 regulates neuron-type-specific genes in a context-dependent and likely indirect manner.

To gain a deeper understanding of how NFYA-1 could regulate neuron-specific features, we focused on VA/VB motor neuron expression of the *del-1* gene.^18^ Our analysis showed that a *del-1* reporter is upregulated in the VA but not the VB neurons in animals lacking NFYA-1. Interestingly, we did not detect an NFYA-1 ChIP-Seq binding peak at the *del-1* locus. Hence, we carefully analyzed previously published regulatory networks that regulate VA neuron expression. We wondered if *del-1* upregulation in *nfya-1(-)* mutants could be due to dysregulation of UNC-3/COE, UNC-4/Prd-type homeodomain protein and UNC-37/Groucho expression, as correct expression of these TFs is essential for correct *del-1* expression in VA/VB neurons.^19–21^ Our data revealed that NFYA-1 is required for correct expression of UNC-3, UNC-4, and UNC-37 in these neurons and thus NFYA-1 likely indirectly controls *del-1* expression by regulating these key TFs. Unfortunately, we were unable to generate compound mutants for *nfya-1*, *unc-3,* and *unc-37* due to lethality. However, *unc-4(-); nfya-1(-)* double mutants do not exhibit an additive effect in *del-1* expression. In fact, this compound mutant exhibits reduced *del-1* levels when compared with *unc-4(-)* single mutant. This could be explained by the lower expression of UNC-3 observed in the *nfya-1* single mutant. These data thus suggest that NFYA-1 regulates *del-1* expression indirectly through the regulation of other transcriptional regulators, such as UNC-3, UNC-37, and UNC-4.

The results detailed above pose an interesting question regarding how NFYA-1 regulates neuron-specific gene batteries. Our data suggest that NFYA-1 can act directly or indirectly to regulate neuron-specific genes that are not bound by NFYA-1. This question will benefit from further studies since one possible explanation could be due to the lack of sensitivity of the whole-animal ChIP-seq technique. Nonetheless, further investigation would be beneficial to better understand how NFYA-1 regulates gene expression in different contexts. One option would be to perform ChIP-seq in specific neurons to increase sensitivity and enhance understanding of the full neuronal regulatory landscape of neuron-specific genes. Another option could be to perform RNA-seq in control and *nfya-1(-)* animals in specific neurons, followed by reporter analysis of the affected genes.

In contrast with neuronal-specific genes, our work revealed that NF-Y appears to have a binding bias toward pan-neuronal promoters (15/15 promoters presented ChIP peaks). These findings made us question: How does NFYA-1 regulate pan-neuronal features in different neuronal subclasses? For this analysis, we measured the expression of fluorescent reporters for genes that encode factors with general functions in synaptic transmission (*rab-3/RAB3, unc-11/SNAP91*, *ric-19/ICA1* and *snb-1/RAB2B*) and neuropeptide processing (*egl-3/PCSK2*). In *nfya-1(-)* animals, we found that *rab-3* and *unc-11* expression is reduced, *snb-1* expression is increased, and no change was detected for *ric-19* or *egl-3* expression. Together with our bioinformatic analysis, these results reveal that the presence of an NFYA-1 binding peak is not sufficient for gene regulation and that additional regulatory factors may cooperate with NFYA-1 to regulate pan-neuronal gene expression.

These results promoted another question that required more in-depth analysis: Does NFYA-1 cooperate with other factors to control pan-neuronal gene expression in different neuronal contexts. By using *rab-3/RAB3* as our model, we aimed to better understand how NFYA-1 controls pan-neuronal gene expression in different neuron subclasses. We hypothesized that NFYA-1 could be a regulator of CUT homeobox gene expression or cooperate with CUT TFs at pan-neuronal loci. Our bioinformatic analysis suggested that NFYA-1 was not a direct regulator of CUT homeobox gene expression. Further, *nfya-1(-)* loss did not affect expression of the CUT TF, CEH-38/ONECUT*.* Together, these results suggest that NFYA-1 cooperates with CUT TFs to regulate *rab-3* expression. At the *rab-3* locus, we found that mutation of the CUT TF binding site did not further exacerbate the reduced *rab-3* expression caused by NFYA-1 loss, suggesting that NFYA-1 and CUT genes function together to regulate *rab-3*. However, additional studies are required to fully understand how the NF-Y complex regulates pan-neuronal genes in different neuronal classes. For example, are these regulatory networks conserved for other pan-neuronal genes, such as *snb-1* or *unc-11*? In addition, what other interactors may cooperate with NF-Y to control pan-neuronal gene expression? These questions could be answered by performing proteomic analysis to identify NFYA-1-interacting proteins.

Finally, we wondered if NFYA-1 control of pan-neuronal gene regulation is conserved in the murine model system. We analyzed NF-YA ChIP-seq data from murine terminally differentiated neurons,^22^ and found that mouse NF-YA also binds to the *Rab3a*, *Rab2b* and *Vamp1* regulatory regions. Thus, to ask whether mammalian NF-YA can control *rab-3/RAB3* expression in *C. elegans*, we expressed murine NF-YA in *C. elegans* neurons. We found that the *rab-3* expression defect of *nfya-1* mutant worms is rescued by murine NF-YA. These results reveal the conserved function of NF-Y in the nervous system in evolutionarily distant species. Hence, our studies provide evidence for deeply conserved functions for ubiquitously expressed TFs in controlling fate acquisition in the nervous system.

## Conclusion

In conclusion, our work showed that NFYA-1 can act either as an enhancer or a repressor of neuronal gene expression.^1^ Our data revealed that NF-Y regulates neuronal-specific features directly and indirectly. For example, NFYA-1 regulates *del-1*/DEG/ENaC in the VA neurons, likely in an indirect manner by controlling the expression of other TFs including UNC-3, UNC-4, and UNC-37. Additionally, NFYA-1 fine-tunes pan-neuronal features by cooperating with other TFs (enhancers and repressors) in a context-dependent manner. However, this regulatory process is highly complex and will require additional studies to determine whether the regulatory mechanisms we identified are conserved in other pan-neuronal genes. Finally, we showed that the regulatory networks we identified may be conserved in mammals, warranting the need for further studies to understand how NF-Y regulates gene expression in the nervous system.
